# A Central Small RNA Regulatory Circuit Controlling Bacterial Denitrification and N_2_O Emissions

**DOI:** 10.1128/mBio.01165-19

**Published:** 2019-08-06

**Authors:** Hannah Gaimster, Claire L. Hews, Ryan Griffiths, Manuel J. Soriano-Laguna, Mark Alston, David J. Richardson, Andrew J. Gates, Gary Rowley

**Affiliations:** a School of Biological Sciences, University of East Anglia, Norwich, United Kingdom; b Earlham Institute, Norwich, United Kingdom; University of Georgia

**Keywords:** *Paracoccus denitrificans*, denitrification, nitrous oxide, sRNA, transcriptional regulation

## Abstract

Global atmospheric loading of the climate-active gas nitrous oxide (N_2_O) continues to increase. A significant proportion of anthropogenic N_2_O emissions arises from microbial transformation of nitrogen-based fertilizers during denitrification, making microbial N_2_O emissions a key target for greenhouse gas reduction strategies. The genetic, physiological, and environmental regulation of microbially mediated N_2_O flux is poorly understood and therefore represents a critical knowledge gap in the development of successful mitigation approaches. We have previously mapped the transcriptional landscape of the model soil-denitrifying bacterium Paracoccus denitrificans. Here, we show that a single bacterial small RNA (sRNA) can control the denitrification rate of P. denitrificans by stalling denitrification at nitrite reduction to limit production of downstream pathway intermediates and N_2_O emissions. Overexpression of sRNA-29 downregulates nitrite reductase and limits NO and N_2_O production by cells. RNA sequencing (RNA-seq) analysis revealed 53 genes that are controlled by sRNA-29, one of which is a previously uncharacterized GntR-type transcriptional regulator. Overexpression of this regulator phenocopies sRNA-29 overexpression and allows us to propose a model whereby sRNA-29 enhances levels of the regulator to repress denitrification under appropriate conditions. Our identification of a new regulatory pathway controlling the core denitrification pathway in bacteria highlights the current chasm in knowledge regarding genetic regulation of this pivotal biogeochemical process, which needs to be closed to support future biological and chemical N_2_O mitigation strategies.

## Abstract (precis)

N_2_O is an important greenhouse gas and a major cause of ozone depletion. Denitrifying bacteria play vital roles in the production and consumption of N_2_O in many environments. Complete denitrification consists of the conversion of a soluble N-oxyanion, nitrate (NO_3_^-^), to an inert gaseous N-oxide, dinitrogen (N_2_). Incomplete denitrification can occur if conditions are prohibitive, for example, under conditions of low soil copper concentrations, leading to emission of N_2_O rather than N_2_. Although enzymatically well characterized, the genetic drivers that regulate denitrification in response to environmental and physiological cues are not fully understood. This study identified a new regulatory sRNA-based control mechanism for denitrification in the model denitrifying bacterium P. denitrificans. Overexpression of this sRNA slows the rate of denitrification. This report highlights that there are gaps in understanding the regulation of this important pathway which need to be filled if strategies for N_2_O mitigation can be rationally and carefully developed.

## INTRODUCTION

Nitrous oxide (N_2_O) is a highly potent greenhouse gas contributing to global radiative forcing (1–3) and is a significant cause of ozone depletion (4). The microbial processes of denitrification and nitrification together are significant contributors to the global N_2_O budget, with nearly one-third of the total contributed by agriculture (5). Soils are responsible for ∼70% of N_2_O annual emissions (2), with a rising proportion resulting from microbial metabolism of nitrogen fertilizers in soils (6). Changes in land use and climate change are predicted to increase N_2_O emissions by between 50% and 150% (7), with anticipated changes in denitrification rates (8).

Denitrifying bacteria play key roles in the synthesis and consumption of N_2_O in many environments. In oxygen (O_2_)-limited environments, these bacteria switch from O_2_ respiration to nitrate (NO_3_^-^) respiration where NO_3_^-^ is converted via nitrite (NO_2_^-^), nitric oxide (NO), and N_2_O to dinitrogen (N_2_). However, as N_2_O continues to be emitted into the atmosphere, this last step in denitrification is not always carried out in synchrony with previous steps, but the reasons for this truncated pathway, in terms of bioenergetics, physiology, and environmental control, are not fully resolved.

Paracoccus denitrificans is a well-studied model denitrifying bacterium found in many terrestrial environments (9). In P. denitrificans, it has been shown that the levels of expression of the Nar, Nir, Nor, and Nos enzymes which catalyze denitrification are regulated, at the level of gene expression, by environmental signals that include nitrate, nitrite, NO, oxygen, and copper. The roles of transcriptional regulators FnrP (fumarate and nitrate reduction protein), NNR (nitrite reductase and nitric oxide reductase regulator), and NarR have previously been reported (10–14), but multiple aspects of the environmental drivers and genetic regulation of denitrification are poorly characterized. Small RNAs (sRNAs) are now considered important components of bacterial regulatory networks (reviewed recently [15]). Recent work in our laboratory identified sRNAs expressed by P. denitrificans under aerobic conditions and under denitrifying N_2_O-producing and -consuming conditions. Importantly, 35% of the sRNAs that we identified were differentially expressed between N_2_O-producing and N_2_O-consuming cultures (16), suggesting that these sRNAs could be candidate regulators of denitrification. The aim of this study was to identify core sRNAs that regulate denitrification in P. denitrificans (specifically, cellular N_2_O emissions). We characterized one of these sRNAs and demonstrated that it is an important regulator of denitrification, where modulation of its levels has impacts on nitrite reduction, resulting in reduced NO and N_2_O emissions. We also found that a previously uncharacterized GntR-type transcriptional regulator is controlled by this sRNA and propose a model whereby this sRNA enhances GntR levels to repress denitrification. This approach has revealed, for the first time, a mechanistic role for sRNA in regulating denitrification in response to key environmental drivers and points to a significant gap in our understanding of this important process.

## RESULTS

### sRNA-29 overexpression reduces N_2_O emissions from denitrifying cultures of P. denitrificans.

**FIG 1:**
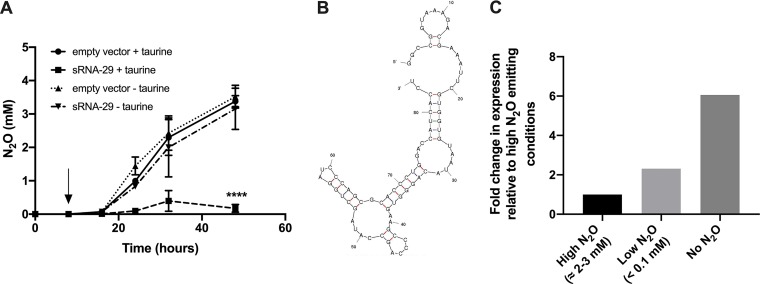
sRNA-29 causes a reduction in the amount of N_2_O produced by P. denitrificans, is predicted to form a complex secondary structure, and is expressed at its highest levels under aerobic conditions in the absence of N_2_O emission. (A) Six cultures of P. denitrificans plus empty pLMB509 vector (circles) and 6 cultures of P. denitrificans plus pLMB509 plus sRNA-29 (squares) were grown under denitrification conditions (20 mM nitrate as electron acceptor) for 8 h. Three of 6 of the cultures then had 10 mM taurine added (indicated with an arrow, circles and squares), while the remaining 3 continued to be incubated in the absence of taurine (triangles). N_2_O levels were measured. Error bars represent standard deviations (SD) of results obtained between triplicate experiments; where not visible, the error bars were smaller than the symbols. ****, *P < *0.0001. (B) The secondary structure was predicted using Mfold. (C) Expression levels are shown relative to the levels expressed under conditions high N_2_O emission as reported previously (16).

We previously characterized the impact that copper and oxygen availability has on the regulation of the denitrification apparatus and subsequent N_2_O emissions (14, 17, 18). Through manipulating these well-characterized parameters, we went on to identify the sRNA complements transcribed by P. denitrificans that are differentially expressed under N_2_O-producing (Cu restriction) and -consuming (Cu repletion) conditions (16). Using this information, we began to screen for sRNAs which, when overexpressed, modulated N_2_O emissions. Cultures of P. denitrificans were grown under batch-denitrifying conditions with nitrate as the electron acceptor for 8 h to establish nitrate-reducing biomass, and then sRNAs were overexpressed in *trans* to determine their impact on N_2_O emissions. From this screen, we selected sRNA-29 for further investigation as, after overexpression of sRNA-29, there was a significant reduction (*P < *0.0001) in the amount of N_2_O emitted for the duration of the experiment compared to the amount seen with empty vector control cultures (Fig. 1A). Here, sRNA-29 was overexpressed from a taurine-inducible promoter in the pLMB509 vector, where addition of 10 mM taurine has previously been reported to induce a 15-fold to 20-fold increase in expression (19). In the presence of 10 mM taurine, approximately 3 mM N_2_O was produced by empty vector control cultures at 48 h compared to ∼0.3 mM N_2_O when sRNA-29 was overexpressed, representing a 10-fold reduction in N_2_O emissions. Where no taurine was added to the empty vector control or to the sRNA-29-containing vector, no significant differences in N_2_O emissions (∼3 mM) were observed. Subsequent experiments were therefore conducted to enable a comparison between the empty vector with taurine and the sRNA-containing vector with taurine. To our knowledge, this represents the first demonstration of a bacterial sRNA impacting N_2_O emissions.

sRNA-29 is an 84-bp-long antisense sRNA encoded on the positive strand of P. denitrificans chromosome 1, transcribed in the orientation opposite that of P. denitrificans 0526 (Pden_0526), which is a nucleoside ABC transporter membrane protein. sRNA-29 is predicted to form a highly complex structure with stem loops as shown in Fig. 1B. sRNA-29 is most highly expressed by P. denitrificans under aerobic conditions (when no N_2_O is produced) and under conditions of low N_2_O emission (when N_2_O is emitted at less than 0.1 mM). In cultures emitting high levels of N_2_O (i.e., when N_2_O is emitted at between 2 and 3 mM), only very low levels of sRNA-29 can be measured, as indicated in Fig. 1C. These expression levels fit with the physiological picture; when *Paracoccus* is forced to produce this sRNA under such conditions of high N_2_O emission, a significant reduction in the N_2_O levels is observed. This further supports the idea of a key regulatory role for this sRNA in controlling condition-appropriate N_2_O production by P. denitrificans.

### sRNA-29 overexpression causes repression of denitrification genes.

**FIG 2:**
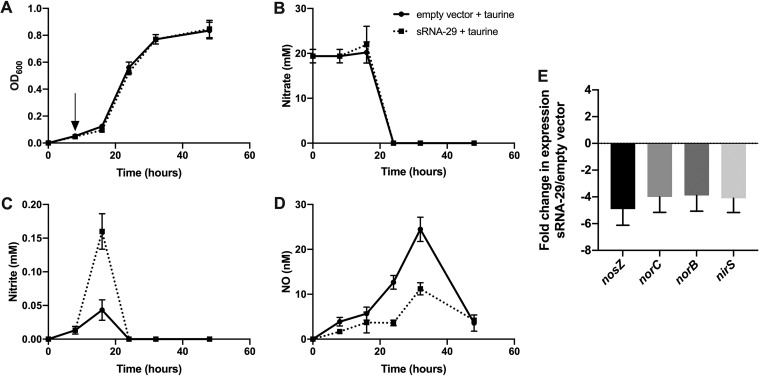
Overexpression of sRNA-29 causes nitrite accumulation and a reduction in the amount of NO produced by P. denitrificans as a consequence of reducing the expression of denitrification genes. P. denitrificans plus empty pLMB509 vector (solid line) or sRNA-29 (dashed line) was grown under denitrification conditions (20 mM nitrate as electron acceptor) for 8 h, and then 10 mM taurine was added (indicated with an arrow) to induce expression of sRNA-29 and levels of growth and denitrification intermediates were measured. (A) Optical density (OD_600_), (B) Nitrate (expressed in millimoles). (C) Nitrite (expressed in millimoles). (D) NO (expressed in nanomoles). (E) qRT-PCR validation of changes in expression of genes involved in denitrification. Error bars represent SD of results obtained between triplicate experiments; where not visible, the error bars were smaller than the symbols.

The clear change in the level of N_2_O flux caused by overexpression of sRNA-29 indicated that the sequential reduction of NO_3_^-^ to N_2_ was in some way perturbed by sRNA-29. To determine the inhibitory effects on the pathway, we measured the levels of all of the denitrification intermediates after overexpression of sRNA-29 (Fig. 2).

Overexpression of sRNA-29 caused no difference in either the growth rate or rate of nitrate reduction by P. denitrificans. Nitrate was reduced from 20 mM to undetectable levels by 24 h, regardless of sRNA-29 overexpression conditions. However, overexpression of sRNA-29 caused a modest but consistent accumulation in the amount of nitrite transiently produced by P. denitrificans. Levels of nitrite in the media accumulated at approximately 0.15 mM when sRNA-29 was overexpressed, representing levels approximately 3-fold higher than the highest level observed in the empty vector control cultures (i.e., 0.01 to 0.05 mM). Nitrite accumulation can have a bacteriostatic action on P. denitrificans (20), but the low levels of accumulation seen here had no effect on the growth of P. denitrificans. Overexpression of sRNA-29 also caused a reduction in the amount of NO produced by P. denitrificans across the experiment, with a maximum level of 10 nM produced when sRNA-29 was overexpressed, compared to 25 nM in the empty vector control culture.

This suggested that sRNA-29 was repressing the expression of the enzymes required for the stage of nitrite reduction in denitrification. To test this, reverse transcription-quantitative PCR (qRT-PCR) was performed on RNA extracted from cultures with and without sRNA-29 overexpression (Fig. 2E). In agreement with the previously obtained phenotypic data, the levels of expression of nitrite reductase gene *nirS* (Pden_2487), the *norB* (Pden_2483) and *norC* (Pden_2484) genes encoding catalytic subunits of nitric oxide reductase, and *nosZ* (Pden_4219) encoding nitrous oxide reductase were all downregulated between 4-fold and 5-fold when sRNA-29 was overexpressed.

### sRNA-29 represses NirS production and nitrite reduction.

**FIG 3:**
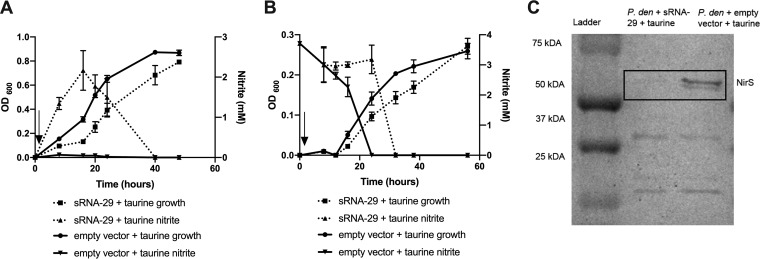
Immediate overexpression of sRNA-29 causes slower growth resulting from nitrite accumulation when grown on nitrate (A) or from slower nitrite consumption when grown on nitrite (B) due to reduced *nirS* expression. P. denitrificans plus empty pLMB509 vector (solid line) or sRNA-29 (dashed line) was grown under denitrifying conditions (with 20 mM nitrate [A] or with 3 mM nitrite [B] as the electron acceptor) with 10 mM taurine added to induce sRNA-29 overexpression at time zero (indicated with an arrow). OD_600_ and nitrite levels were measured. Error bars represent the SD of results of comparisons between triplicate experiments, and where not visible, the bars are smaller than the symbols. (C) Soluble protein was extracted from both cultures at 24 h and subjected to SDS-PAGE and subsequent heme staining. *P. den*, P. denitrificans.

The nitrite accumulation observed in cultures of P. denitrificans in which sRNA-29 had been overexpressed 8 h into growth suggested an initial bottleneck in denitrification at nitrite reduction. We sought to explore the mechanism of sRNA-29 control further under different conditions. First, overexpression of sRNA-29 was induced at the start of growth in anaerobic nitrate-reducing cultures rather than, as in previous experiments, after biomass had accumulated. This immediate induction of sRNA-29 lead to slower growth of P. denitrificans, with the optical density at 600 nm (OD_600_) of the sRNA-29 overexpression culture approximately 2-fold lower than that seen with the empty vector control culture at time points between 16 and 40 h (Fig. 3A). This corresponded precisely to the large accumulation of nitrite observed in these cultures at this time point, with sRNA-29 overexpression causing an accumulation of 2 mM nitrite by 16 h, compared to an accumulation of approximately 0.02 mM in the control cultures. It is likely that this 100-fold-higher accumulation of nitrite seen when sRNA-29 was overexpressed in cultures led to the observed slower growth. These data pointed to a significant inhibitory effect of sRNA-29 on nitrite reduction. We investigated this further by determining the impact of sRNA-29 overexpression on the ability of P. denitrificans to utilize nitrite as the electron acceptor. Here, overexpression of sRNA-29 also caused slower growth on nitrite (Fig. 3B), due to impaired ability of P. denitrificans to reduce nitrite in the presence of sRNA-29. Previous data showed that overexpression of sRNA-29 resulted in a reduction in the expression level of *nirS* mRNA (Fig. 2E), and we sought to confirm the impact of this on NirS protein levels under these conditions. Soluble protein was extracted from cultures of P. denitrificans in which sRNA-29 was overexpressed and also from the empty vector control cultures at 24 h. Analysis by heme-staining SDS-PAGE (Fig. 3C) revealed relative differences in the expression of heme-containing proteins; the levels were then determined by densitometric analyses. The level of NirS, representing a 68-kDa heme-staining band (11), measured under conditions of sRNA-29 overexpression was approximately 9-fold lower than that seen with the empty vector control. This reduction in the protein level was specific to NirS, as the levels of other heme-containing proteins were very similar across the two conditions.

### Mutation of sRNA-29 to disrupt secondary structure abolishes its control on denitrification.

**FIG 4:**
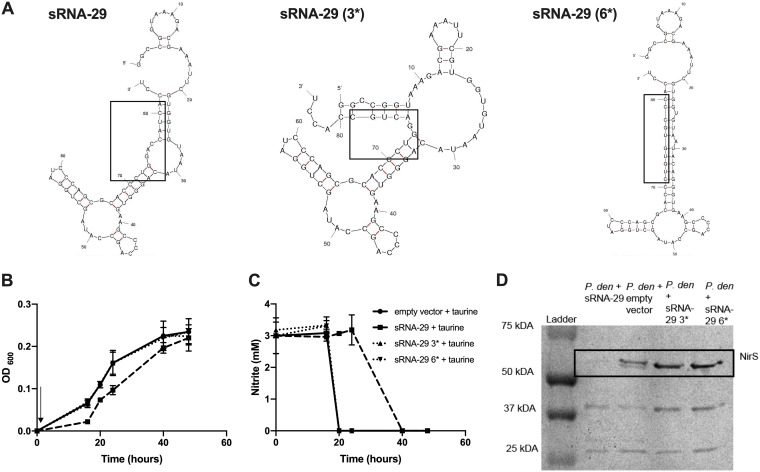
Mutation of either 3 or 6 nucleotides of sRNA-29 alters the predicted secondary structure, which results in a loss of function under conditions of growth on nitrite. (A) Mutation of 3 (3*) or 6 (6*) nucleotides of sRNA-29 altered the secondary structure as predicted by Mfold. P. denitrificans plus empty pLMB509 vector (solid line) or sRNA-29 or sRNA-29 with 3 (3*) or 6 (6*) nucleotides mutated (various dashed lines and symbols as indicated in legend) was grown under denitrification conditions (3 mM nitrite as electron acceptor) with 10 mM taurine added to induce sRNA-29 overexpression at time zero (indicated with an arrow in panel B). OD_600_ levels (B) and nitrite consumption levels (C) were measured. Error bars represent SD of results of comparisons between triplicate experiments, and where not visible, the bars were smaller than the symbols. (D) Soluble protein was extracted from cultures at 24 h and subjected to SDS-PAGE and subsequent heme staining.

In order to demonstrate that the impact on denitrification was a direct effect of sRNA-29, we mutated either a 3-bp region or 6-bp region between bases 72 and 80 to disrupt the secondary structure (shown in Fig. 4). Nitrite consumption levels during growth and NirS levels were measured when these mutated versions of sRNA-29 were overexpressed. The mutants had no impact on growth or nitrite consumption compared to the empty control, whereas the wild-type (WT) sRNA caused slower growth, presumably due to slower nitrite consumption.

The mutated sRNA also had no impact on the levels of cellular NirS compared to the empty control, whereas the WT sRNA reduced the levels significantly. This suggests that the secondary structure of sRNA-29 is important for its function regarding denitrification, as its function is abolished when it is perturbed by small base pair substitutions.

### Overexpression of sRNA-29 changes the expression levels of 53 genes across the P. denitrificans genome.

**FIG 5:**
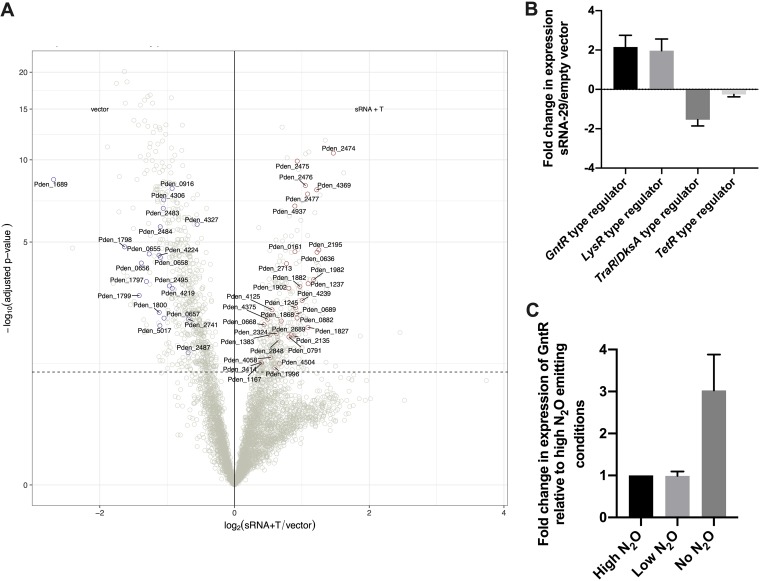
Overexpression of sRNA-29 changes the expression of 53 genes across the P. denitrificans genome. (A) Volcano plot showing upregulated (red) and downregulated (blue) transcripts (*P* < 0.05) from RNA-seq data under conditions of overexpression of sRNA-29 in the presence of taurine (*n* = 3) versus the empty vector in the presence of taurine (*n* = 3). The dashed horizontal line represents an adjusted *P* value of 0.05. (B and C) qRT-PCR validation of changes in expression of genes of interest under conditions of sRNA-29 plus empty vector (B) or under conditions of high levels of NO_2_ emission.

As there is no nucleotide sequence homology between sRNA-29 and *nirS* mRNA or the 5′ untranslated region (5′ UTR) to act as a seed region, there had to be at least another component of the sRNA-29-controlled regulatory pathway. An obvious candidate for this would be the nitric oxide reductase regulator (NNR), a well-characterized transcriptional regulator of *nirS*, but, again, there is no sequence homology between sRNA-29 and *nnr*. In order to identify the missing components of the regulatory cascade and systematically identify targets of sRNA-29 across the whole genome of P. denitrificans, we combined sRNA-29 overexpression with RNA sequencing (RNA-seq). Here, we reverted to the use of nitrate as the electron acceptor with sRNA-29 overexpression at 8 h to avoid any impact of differential growth rates on transcription between the sRNA-29 overexpression and control cultures. As shown in the volcano plot in Fig. 5A, we observed at least 2-fold change in the expression levels of 53 genes in response to sRNA-29 overexpression (*P = *0.05) when the results were normalized to those seen with the empty vector control cultures. This demonstrates that these 53 genes are subject to either direct or indirect regulation by sRNA-29. The full list is also contained in Table S1 in the supplemental material. As validation of the RNA-seq experimental design and data, genes encoding the denitrification apparatus, after nitrate reduction, were downregulated in response to sRNA-29, in agreement with the previously obtained qRT-PCR data. Specifically, nitrite reductase *nirS* (Pden_2487) was downregulated by a log2-fold change of −1.04. The genes encoding the catalytic subunits of nitric oxide reductase, *norB* (Pden_2483) and *norC* (Pden_2484), were downregulated by log2-fold changes of −1.07 and −1.08, respectively, and the gene encoding nitrous oxide reductase, *nosZ* (Pden_4219), was downregulated by a log2-fold change of −1.02. The 53 differentially expressed genes were sorted into functional categories, with most (17/53) assigned as encoding hypothetical proteins of unknown function. Among the most common gene product categories regulated by sRNA-29 were proteins involved in energy metabolism (9/53); examples included phosphoadenylylsulfate reductase (Pden_0656) and ferredoxin-NADP(+) reductase (Pden_0658). The levels of expression of all nine of these targets were downregulated in response to sRNA-29. Other common categories included genes involved in transport (10/53); examples included those encoding an efflux transporter (Pden_0161) and a molybdenum ABC transporter (Pden_1167). These showed variable changes in expression levels, with five of the genes upregulated and the other five downregulated in response to sRNA-29. Eight genes involved in carbohydrate metabolism were all upregulated in response to sRNA-29; examples included genes encoding 6-phosphofructokinase (Pden_0882) and 2-keto-3-deoxy-phosphogluconate aldolase (Pden_1245). However, perhaps most intriguingly, the expression levels of genes encoding four transcriptional regulators were also altered in response to sRNA-29, with two being downregulated (those encoding a TetR family regulator [Pden_0668] and a TraR/DksA family regulator [Pden_0916]) and two being upregulated (those encoding a GntR-type regulator [Pden_2475] and a LysR family regulator [Pden_4369]) (these changes were validated using qRT-PCR as shown in Fig. 5B). All four regulators were previously uncharacterized in P. denitrificans, or with regard to denitrification, and may well act as the missing node in sRNA-29 regulation of *nirS*.

10.1128/mBio.01165-19.1 TABLE S1 Full list of genes regulated by sRNA-29. Download Table S1, PDF file, 0.1 MB. Copyright © 2019 Gaimster et al. 2019 Gaimster et al. This content is distributed under the terms of the Creative Commons Attribution 4.0 International license.

### sRNA-29 works via a novel GntR-type transcription factor to control denitrification.

**FIG 6:**
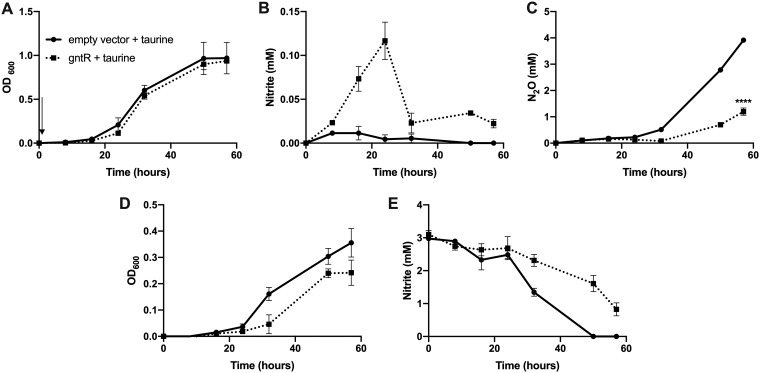
Overexpression of GntR phenocopies overexpression of sRNA-29 under conditions of growth on nitrite. P. denitrificans plus empty pLMB509 vector (solid line) or pLMB509 with *gntR* cloned into the overexpression site (dashed line) was grown under denitrification conditions with either 20 mM nitrate or 3 mM nitrite as the electron acceptor with 10 mM taurine added to induce GntR overexpression at time zero (indicated with an arrow). OD_600_ (A), nitrite accumulation (B), and N_2_O production (C) were measured under conditions of growth on nitrate, and OD_600_ (D) and nitrite consumption (E) were measured under conditions of growth on nitrite. Error bars represent SD of results of comparisons between triplicate experiments, and where not visible, the bars were smaller than the symbols (*P < *0.0001).

Among the four transcriptional regulators which showed differential levels of expression in response to sRNA-29 in the RNA-seq analysis, we noted that the GntR-type regulator identified displayed conserved expression patterns identical to those seen with sRNA-29 under the various conditions that we had tested previously (16). We also verified these data using qRT-PCR (Fig. 5C), and the results confirmed that, like sRNA-29, the GntR-type regulator was most highly expressed under aerobic conditions (no N_2_O), with lower levels of expression measured from cultures with low and high levels of N_2_O emission, respectively. Additionally, of the 4 regulators differentially expressed in response to sRNA-29, only the GntR-type regulator shares a 7-bp region of sequence homology, located within the coding DNA sequence (CDS), making this the best candidate for identification as a putative repressor of *nirS* transcription. In order to see if sRNA-29 was mediating its effect via this transcriptional regulator, we overexpressed the GntR-type regulator and monitored levels of growth and nitrite reduction with either nitrate or nitrite as the electron acceptor (Fig. 6). The results showed that there was no significant difference in growth levels when the GntR-type regulator was overexpressed when P. denitrificans was grown on nitrate but that there was a significantly higher level of nitrite accumulation when the GntR-type regulator was overexpressed. Levels of nitrite in the media accumulated at approximately 0.12 mM when GntR was overexpressed, compared to levels of approximately 0.01 to 0.05 mM in the empty vector control cultures. This approximately 3-fold change was almost identical to the fold change in nitrite accumulation observed when sRNA-29 was expressed 8 h into growth (Fig. 2), which also was not sufficient to slow the growth.

Overexpression of the GntR-type regulator also significantly reduced the amount of N_2_O produced by the cultures (*P < *0.0001). Approximately 3 mM N_2_O was produced by empty vector control cultures at 48 h, compared to approximately 0.5 mM N_2_O produced when the GntR-type regulator was overexpressed. Furthermore, under conditions of growth on nitrite, GntR-type regulator overexpression resulted in a reduction in the growth rate, with the OD_600_ of the GntR-type regulator overexpression culture lower than the OD_600_ of the empty vector control culture at time points between 24 and 48 h. This was likely caused by the reduced efficacy of nitrite consumption observed over this time period. Taken together, these data show that GntR-type regulator overexpression phenocopies sRNA-29 overexpression, at the level of nitrite reduction, and that this newly identified GntR-type transcription factor likely functions with sRNA-29 as a repressor of denitrification.

## DISCUSSION

Denitrification is an important factor in the bacterially driven flux of N_2_O to the atmosphere. A comprehensive understanding of the molecular mechanisms and environmental drivers that underpin denitrification is lacking, which is surprising given the importance of this biogeochemical cycle. Here, we report a novel mechanism of regulation of denitrification via a bacterial sRNA. The data presented here suggest a model whereby sRNA-29 acts to stabilize expression of a previously unknown GntR-type transcriptional regulator, which in turn represses the rate of denitrification. This model fits well with the observed expression patterns of sRNA-29; it is expressed most highly under aerobic conditions, where we hypothesize it contributes to the repression of denitrification genes under those conditions. Conversely, it is expressed at lowest levels when complete denitrification is occurring (i.e., under conditions of low levels of N_2_O emission). The computationally predicted 7-bp seed is located within the CDS of *gntR*. On the basis of our physiological and expression data, we hypothesize a mechanism by which sRNA-29 stabilizes *gntR* mRNA, perhaps through blocking the activity of RNase-mediated decay. Future experimental evaluation of the interactions of the sRNA with the mRNA (and of the role of any RNA chaperone) will explore the detailed molecular interactions of the regulatory model. On the basis of our findings, we propose that sRNA-29 should now be referred to as *denR* (denitrification repressor) and that the GntR-type transcriptional regulator should now be called NirR (nitrite reductase repressor).

This is, to our knowledge, the first example of a sRNA directly regulating denitrification. More widely, there is some precedent for the role of sRNA in nitrogen metabolism in general terms. For example, the SdsN sRNA contributes to nitrogen metabolism in *Salmonella*, via regulation of the nitrate- and nitrite-responsive NarP transcription factor (21). Studies have also described the sRNA landscape in other bacterial denitrifiers, perhaps most notably in Pseudomonas aeruginosa, but mainly in the context of virulence (22). More recently, a sRNA was described in a clinical isolate of P. aeruginosa 14 (PA14) which seemed to be important for denitrification. However, overexpression of the sRNA did not affect levels of nitrite reductase, leading the authors to conclude that this sRNA was exerting an indirect effect on denitrification (23), in comparison to the sRNA described in this study.

Importantly, sRNA-29 is well conserved across multiple denitrifying species of bacteria in the *Rhodobacteraceae* genus, including closely related species such as Paracoccus aminophilus but also in more distantly related bacteria such as the marine denitrifier Ruegeria pomeroyi (16). Additionally, BLASTn searches indicated that, among the species predicted to encode sRNA-29 homologues, all of these also encode a transcriptional regulator with homology to the GntR-type regulator which is the target of sRNA-29 in P. denitrificans. This may suggest a conserved mechanism by which sRNA-29 is able to mediate impact on denitrification rates via this regulator, in a wide range of terrestrial and marine environments. Accordingly, this strongly suggests that sRNAs are common and yet uncharacterized nodes in regulation of denitrification that have the potential to be important new targets for controlling cellular production and emission of N_2_O.

The GntR-type regulator identified in this work (NirR) was not previously known to be involved in denitrification in P. denitrificans. GntR-type transcriptional regulators are found across many bacterial species and play key roles in regulating a wide array of processes. This type of transcriptional regulator consists of a conserved N-terminal HTH (helix-turn-helix) DNA-binding domain linked to a variable C-terminal signaling domain (24). Future work is required to understand the entire NirR regulon and stimulating cues.

In conclusion, this identification of a previously unappreciated regulatory pathway controlling denitrification demonstrates the significant gaps in our understanding of this process, which need to be filled if realistic novel field strategies are to be developed that can help inform future development of N_2_O mitigation. We propose that by uncovering the sRNAs which alter N_2_O emissions, it could be possible to utilize these sRNA to specifically target and knock down N_2_O emissions from P. denitrificans. Clearly, this has been an understudied area to date; however, this report marks the initial foray into this field. It will be important that, in the future, work on model organisms such as P. denitrificans is completed alongside meta-analyses of sRNA in the environment, as well as of the role of sRNA in *nosZ* clade II-carrying organisms to potentially drive environments to be N_2_O sinks (25). Furthermore, there are many other sRNAs which have shown differential levels of expression across different N_2_O emission conditions in P. denitrificans (16) and we suggest that this is the start of a stepwise change in the understanding of regulation of denitrification and flux of N_2_O.

## MATERIALS AND METHODS

### Bacterial strains and growth media and conditions.

Paracoccus denitrificans was grown in a defined minimal medium which contained 29 mmol/liter Na_2_HPO_4_, 11 mmol/liter KH_2_PO_4_, 10 mmol/liter NH_4_Cl, and 0.4 mmol/liter MgSO_4_ and which was supplemented with 30 mmol/liter sodium succinate, 20 mmol/liter NaNO_3_ or 3 mmol/liter NaNO_2_, and 2 ml/liter Vishniac and Santer trace elements solution [130 mmol/liter EDTA, 7.64 mmol/liter ZnSO_4_, 25 mmol/liter MnCl_2_, 18.5 mmol/liter FeSO_4_, 0.89 mmol/liter (NH_4_)_6_Mo_7_O_24_, 6.4 mmol/liter CuSO_4_, 6.72 mmol/LCoCl_2_, 37.4 mmol/liter CaCl_2_]. N_2_O emission culture conditions were created by omitting CuSO_4_ from the trace elements solution as described previously (14). Anaerobic batch cultures (200 ml) were inoculated with a 1% (vol/vol) concentration of stationary-phase cells that had been previously grown in minimal media. The vessels used were 250-ml Duran bottles with screw-cap lids and gas-tight silicone septa. Cultures were sparged with N_2_ for 10 min to impose an anoxic environment and incubated statically at 30°C. Taurine was added to induce expression of sRNA at a final concentration of 10 mM.

### Genetic manipulation of P. denitrificans.

The 84-bp sequence of sRNA-29 or the GntR-type regulator was cloned into the overexpression vector, pLMB509 (19), at the first NdeI site using the gene synthesis service from GenScript to produce pLMB509-sRNA-29 and pLMB509-GntR. Plasmids were transformed into P. denitrificans by the use of triparental mating as previously described (14).

### Measurement of OD_600_ and N_2_O, NO, NO_2_^-^, and NO_3_^-^ levels.

Headspace gas samples (3 ml) were taken using a 5-ml gas-tight syringe (Hamilton) and stored in 3-ml preevacuated screw cap Exetainer vials (Labco). N_2_O gas samples were analyzed by gas chromatography (GC) through injection of a 50-μl sample into a Clarus 500 gas chromatographer (PerkinElmer) with an electron capture detector and Elite-Plot Q (DVB plot column, 30 m by 0.53 mm inner diameter [ID]; carrier, N_2_; inert portion, 95% [vol/vol] argon–5% [vol/vol] methane). Standards of N_2_O (Scientific and Technical Gases) (5, 100, 1,000, 5,000, and 10,000 ppm) were used to quantify N_2_O levels. Total N_2_O amounts were calculated by applying Henry’s Law constant (*K_H_*) for N_2_O at 30°C (*K_H_^cc^* of 0.5392). Values of N_2_O (in micromoles) were multiplied by 2 to adjust values to micromoles of N in the form of N_2_O (N⋅N_2_O); this takes into account of the stoichiometry of N in N_2_O. NO levels in the headspace of Duran bottles (200 ml) were measured using a nitric oxide analyzer (NOA) (model 280i; General Electric).

Liquid aliquots (1 ml) were sampled for each culture using a needle and syringe. The optical density (λ 600 nm) was measured using a SpectraMax M5 spectrophotometer. The sample was centrifuged at 16,000 × *g* for 5 min to pellet residual cells, and the supernatant was stored at −20°C. NO_3_^-^ and NO_2_^-^ concentrations were measured using the NOA, with 20 μl of thawed liquid sample being injected into the purge vessel. The purge vessel contained 3 ml of either NaI (1% [wt/vol] in acetic acid) for analysis of NO_2_^-^ or VCl_3_–1% HCl for analysis of NO_3_^-^ and was connected to a chemiluminescence detector. The reducing agent was continuously subjected to N_2_ agitation in order to transport NO through the NOA and to maintain an anoxic environment.

### RNA extraction.

Each experiment was performed under each set of conditions in 3 biological repeats, and RNA was extracted from these cultures. For RNA extraction, 30 ml of mid-exponential-phase cells (OD_600_ of approximately 0.4) was added to 12 ml of ice-cold 95% ethanol–5% phenol (vol/vol) solution (pH = 4.3) and the reaction mixture incubated on ice for 30 min to stabilize RNA and prevent degradation. RNA was isolated using the TRIzol method according to a previously described protocol (26). Trace DNA contamination was removed using Turbo DNA-free DNase (Ambion), and successful removal of DNA contamination was confirmed by PCR amplification of RNA samples using MyFi DNA polymerase (Bioline) according to the manufacturer’s instructions. RNA was quantified spectrophotometrically using a NanoDrop 2000 system (Thermo Scientific), and the integrity of the RNA samples was analyzed using an Experion automated electrophoresis platform (Bio-Rad) and RNA StdSens chips (Bio-Rad) according to the manufacturer’s instructions.

### Library preparation and sequencing.

Library preparation and sequencing were performed by the Wellcome Trust Centre for Human Genetics, Oxford. The rRNA-depleted fraction was selected from the total RNA provided before conversion to cDNA. Second-strand cDNA synthesis incorporated dUTP. The cDNA was then end repaired, A tailed, and adapter ligated. Prior to amplification, samples underwent uridine digestion. The prepared libraries were size selected and multiplexed and subjected to quality control (QC) before 75-bp paired-end sequencing was performed over one lane of a flow cell. Data were aligned to the reference genome and subjected to quality checks. Standard data files were provided as fastq and bam files. Quality control and adapter trimming were applied to the 368 million raw 75-bp paired-end Illumina reads using TrimGalore v0.4.2 with the parameters –q 20, –length 30, and –stringency 5 (https://www.bioinformatics.babraham.ac.uk/projects/trim_galore/). On average, ∼0.01% of reads were removed per sample. Alignment-free mapping and quantification of the reads with respect to the reference transcriptome for Paracoccus denitrificans PD1222 were carried out using Kallisto v0.43.0; a k-mer size of 31 was used to build the transcriptome index, and the quantification procedure used 100 bootstraps per sample ((27)). The reference transcriptome was obtained from Ensembl Bacteria as cDNA sequences in FASTA format. The R (v3.4.0) statistical software environment was used for the RNA-seq analyses. The bioconductor packages used were tximport v1.4.0 (28) (to summarize Kallisto’s transcript-level quantification as matrices of counts) and DESeq2 v1.16.0 (29) (to analyze the differential expression levels of transcripts between conditions). The R packages ggplot2, ggrepel (https://CRAN.R-project.org/package=ggrepel), and dplyr (https://CRAN.R-project.org/package=dplyr) were used for generating plots.

### qRT-PCR.

Total RNA from P. denitrificans (2 μg) was reverse transcribed to cDNA using SuperScript II reverse transcriptase and random primers (Invitrogen) in a final volume of 20 μl, according to the manufacturer’s specifications. Following reverse transcriptase reactions, cDNA was diluted 1:5 with H_2_O before use in quantitative PCRs. Primers were designed to amplify products of between 100 and 150 bp, with a melting temperature (*T_m_*) of ∼60°C, and were used at a final concentration of 0.4 μmol/liter (listed in Table S1 in the supplemental material). Real-time quantification of transcripts was done using a SensiFAST SYBR No-ROX kit (Bioline), a C1000 thermal cycler, and a CFX96 real-time PCR detection system (Bio-Rad), according to the specifications of the manufacturers. Each reaction was performed in triplicate, and transcripts were quantified from three RNA preparations isolated from independent biological replicates. Standard curves and amplification efficiencies were determined using serially (10-fold) diluted genomic DNA from a stock concentration of 100 ng/μl. The relative fold change values were calculated using amplification efficiencies, as described previously. Transcript abundance was normalized to *dnaN*, encoding the β-subunit of DNA-polymerase III (Pden_0970). Primers (sequences [5′ to 3′]) were as follows: nosZ_forward (CTT TTC GAC CTC CTA CAA CTC), nosZ_reverse (CCG TTC AGT TCC TGA TAG TCG), norB_forward (GTA AAG CCA TTT CTC GAC C), norB_reverse (CTC TTT GCC TTC TAC AAC C), norC_forward (CCC TCG GTC GTC GAG GGC AA), norC_reverse (CCC TCG GTC GTC GAG GGC AA), nirS_forward (TCA ATA TGA TCG ACC TTT GGA T), nirS_reverse (ATC GGC TCC AGC GTC TCG CCG T), GntR_forward (CGC ATT TCG GCC GCG AAC C), and GntR_reverse (CAG CAT CAG CCC TCC GCC GC).

### Preparation of soluble protein, SDS-PAGE, and heme staining.

Cell pellets from 1 l P. denitrificans isolates grown with 3 mM NaNO_2_ as the electron acceptor were harvested by centrifugation at 24 h into the growth period. Pellets were washed twice in 50 mM Tris-HCl at pH 8.0 and resuspended in 1 ml of the same buffer. After addition of MnCl_2_ to reach a concentration of 10 mM and DNase I to reach a concentration of 2 mg ml^−1^, cell extracts were prepared using a French pressure cell (each sample was passed through the French pressure cell 3 times to ensure complete lysis). After removal of the cell debris by centrifugation for 5 min at 20,000 × *g*, the supernatant was centrifuged at 100,000 × *g* for 60 min. The pellet was resuspended in 50 mM Tris-HCl at pH 8.0. Protein concentrations were determined using a bicinchoninic acid (BCA) kit (Pierce) with bovine serum albumin (BSA) as a standard. SDS-PAGE was carried out using a Bio-Rad Mini-Protean II gel system and TruPAGE precast gels. Samples normalized for protein amounts (100 mg of protein each) were diluted in sample buffer (6 M urea, 5% SDS, 10% glycerol, 0.05% bromophenol blue) and were not boiled but were left at room temperature for 10 min to prevent the loss of heme c. The gels were stained for covalently bound heme by addition of 0.25 M sodium acetate with shaking for 15 min. N,N,N',N'-tetramethyl-1,3-butanediamine (TMBD) (1 mg/ml) was added for 1 h, before addition of 200 μl 30% H_2_O_2_. Densitometry analyses were performed using ImageLab software.

## References

[1] Richardson D, Felgate H, Watmough N, Thomson A, Baggs E 2009 Mitigating release of the potent greenhouse gas N2O from the nitrogen cycle—could enzymic regulation hold the key? Trends Biotechnol 27:388–397. doi:10.1016/j.tibtech.2009.03.009. 19497629

[2] Skiba U, Jones SK, Dragosits U, Drewer J, Fowler D, Rees RM, Pappa VA, Cardenas L, Chadwick D, Yamulki S, Manning AJ 2012 UK emissions of the greenhouse gas nitrous oxide. Philos Trans R Soc Lond B Biol Sci 367:1175–1185. doi:10.1098/rstb.2011.0356. 22451103 PMC3306628

[3] Smith KA, Mosier AR, Crutzen PJ, Winiwarter W 2012 The role of N2O derived from crop-based biofuels, and from agriculture in general, in Earth's climate. Philos Trans R Soc Lond B Biol Sci 367:1169–1174. doi:10.1098/rstb.2011.0313. 22451102 PMC3306623

[4] Ravishankara AR, Daniel JS, Portmann RW 2009 Nitrous oxide (N2O): the dominant ozone-depleting substance emitted in the 21st century. Science 326:123–125. doi:10.1126/science.1176985. 19713491

[5] Kroeze C, Bouwman L 2011 The role of nitrogen in climate change. Curr Opin Environ Sustain 3:279–280. doi:10.1016/j.cosust.2011.08.015.

[6] Butterbach-Bahl K, Baggs EM, Dannenmann M, Kiese R, Zechmeister-Boltenstern S 2013 Nitrous oxide emissions from soils: how well do we understand the processes and their controls? Philos Trans R Soc Lond B Biol Sci 368:20130122. doi:10.1098/rstb.2013.0122. 23713120 PMC3682742

[7] Trost B, Prochnow A, Drastig K, Meyer-Aurich A, Ellmer F, Baumecker M 2013 Irrigation, soil organic carbon and N2O emissions. A review. Agron Sustain Dev 33:733–749. doi:10.1007/s13593-013-0134-0.

[8] Fowler D, Steadman CE, Stevenson D, Coyle M, Rees RM, Skiba UM, Sutton MA, Cape JN, Dore AJ, Vieno M, Simpson D, Zaehle S, Stocker BD, Rinaldi M, Facchini MC, Flechard CR, Nemitz E, Twigg M, Erisman JW, Butterbach-Bahl K, Galloway JN 2015 Effects of global change during the 21st century on the nitrogen cycle. Atmos Chem Phys 15:13849–13893. doi:10.5194/acp-15-13849-2015.

[9] Spiro S 2017 Regulation of denitrification, p 312–330. *In* Metalloenzymes in denitrification: applications and environmental impacts. The Royal Society of Chemistry, Cambridge, United Kingdom.

[10] Van Spanning RJM, De Boer APN, Reijnders WNM, Westerhoff HV, Stouthamer AH, Van Der Oost J 1997 FnrP and NNR of Paracoccus denitrificans are both members of the FNR family of transcriptional activators but have distinct roles in respiratory adaptation in response to oxygen limitation. Mol Microbiol 23:893–907. doi:10.1046/j.1365-2958.1997.2801638.x. 9076727

[11] Van Spanning RJM, De Boer APN, Reijnders WNM, Spiro S, Westerhoff HV, Stouthamer AH, Van der Oost J 1995 Nitrite and nitric oxide reduction in Paracoccus denitrificans is under the control of NNR, a regulatory protein that belongs to the FNR family of transcriptional activators. FEBS Lett 360:151–154. doi:10.1016/0014-5793(95)00091-M. 7875319

[12] Bergaust L, van Spanning RJM, Frostegård Å, Bakken LR 2012 Expression of nitrous oxide reductase in Paracoccus denitrificans is regulated by oxygen and nitric oxide through FnrP and NNR. Microbiology 158:826–834. doi:10.1099/mic.0.054148-0. 22174385 PMC3541799

[13] Lee Y-Y, Shearer N, Spiro S 2006 Transcription factor NNR from Paracoccus denitrificans is a sensor of both nitric oxide and oxygen: isolation of nnr* alleles encoding effector-independent proteins and evidence for a haem-based sensing mechanism. Microbiology 152:1461–1470. doi:10.1099/mic.0.28796-0. 16622062

[14] Sullivan MJ, Gates AJ, Appia-Ayme C, Rowley G, Richardson DJ 2013 Copper control of bacterial nitrous oxide emission and its impact on vitamin B12-dependent metabolism. Proc Natl Acad Sci U S A 110:19926–19931. doi:10.1073/pnas.1314529110. 24248380 PMC3856849

[15] Dutta T, Srivastava S 2018 Small RNA-mediated regulation in bacteria: a growing palette of diverse mechanisms. Gene 656:60–72. doi:10.1016/j.gene.2018.02.068. 29501814

[16] Gaimster H, Chalklen L, Alston M, Munnoch JT, Richardson DJ, Gates AJ, Rowley G 2016 Genome-wide discovery of putative sRNAs in Paracoccus denitrificans expressed under nitrous oxide emitting conditions. Front Microbiol 7:1806. doi:10.3389/fmicb.2016.01806. 27895629 PMC5107571

[17] Felgate H, Giannopoulos G, Sullivan MJ, Gates AJ, Clarke TA, Baggs E, Rowley G, Richardson DJ 2012 The impact of copper, nitrate and carbon status on the emission of nitrous oxide by two species of bacteria with biochemically distinct denitrification pathways. Environ Microbiol 14:1788–1800. doi:10.1111/j.1462-2920.2012.02789.x. 22642644

[18] Giannopoulos G, Sullivan MJ, Hartop KR, Rowley G, Gates AJ, Watmough NJ, Richardson DJ 2017 Tuning the modular Paracoccus denitrificans respirome to adapt from aerobic respiration to anaerobic denitrification. Environ Microbiol 19:4953–4964. doi:10.1111/1462-2920.13974. 29076595

[19] Tett AJ, Rudder SJ, Bourdès A, Karunakaran R, Poole PS 2012 Regulatable vectors for environmental gene expression in Alphaproteobacteria. Appl Environ Microbiol 78:7137–7140. doi:10.1128/AEM.01188-12. 22820336 PMC3457481

[20] Hartop KR, Sullivan MJ, Giannopoulos G, Gates AJ, Bond PL, Yuan Z, Clarke TA, Rowley G, Richardson DJ 2017 The metabolic impact of extracellular nitrite on aerobic metabolism of Paracoccus denitrificans. Water Res 113:207–214. doi:10.1016/j.watres.2017.02.011. 28214776 PMC5339346

[21] Hao Y, Updegrove TB, Livingston NN, Storz G 2016 Protection against deleterious nitrogen compounds: role of σ(S)-dependent small RNAs encoded adjacent to sdiA. Nucleic Acids Res 44:6935–6948. doi:10.1093/nar/gkw404. 27166377 PMC5001591

[22] Gómez-Lozano M, Marvig RL, Molin S, Long KS 2012 Genome-wide identification of novel small RNAs in Pseudomonas aeruginosa. Environ Microbiol 14:2006–2016. doi:10.1111/j.1462-2920.2012.02759.x. 22533370

[23] Tata M, Amman F, Pawar V, Wolfinger MT, Weiss S, Häussler S, Bläsi U 2017 The anaerobically induced sRNA PaiI affects denitrification in Pseudomonas aeruginosa PA14. Front Microbiol 8:2312. doi:10.3389/fmicb.2017.02312. 29218039 PMC5703892

[24] Suvorova IA, Korostelev YD, Gelfand MS 2015 GntR family of bacterial transcription factors and their DNA binding motifs: structure, positioning and co-evolution. PLoS One 10:e0132618. doi:10.1371/journal.pone.0132618. 26151451 PMC4494728

[25] Hallin S, Philippot L, Loffler FE, Sanford RA, Jones CM 10 8 2017, posting date. Genomics and ecology of novel N2O-reducing microorganisms. Trends Microbiol doi:10.1016/j.tim.2017.07.003. 28803698

[26] Kröger C, Dillon SC, Cameron ADS, Papenfort K, Sivasankaran SK, Hokamp K, Chao Y, Sittka A, Hébrard M, Händler K, Colgan A, Leekitcharoenphon P, Langridge GC, Lohan AJ, Loftus B, Lucchini S, Ussery DW, Dorman CJ, Thomson NR, Vogel J, Hinton J 2012 The transcriptional landscape and small RNAs of Salmonella enterica serovar Typhimurium. Proc Natl Acad Sci U S A 109:E1277–E1286. doi:10.1073/pnas.1201061109. 22538806 PMC3356629

[27] Bray NL, Pimentel H, Melsted P, Pachter L 2016 Near-optimal probabilistic RNA-seq quantification. Nat Biotechnol 34:525–527. doi:10.1038/nbt.3519. 27043002

[28] Soneson C, Love MI, Robinson MD 2015 Differential analyses for RNA-seq: transcript-level estimates improve gene-level inferences. F1000Res 4:1521. doi:10.12688/f1000research.7563.1. 26925227 PMC4712774

[29] Love MI, Huber W, Anders S 2014 Moderated estimation of fold change and dispersion for RNA-seq data with DESeq2. Genome Biol 15:550. doi:10.1186/s13059-014-0550-8. 25516281 PMC4302049

