# Supplementary material for: A Central Small RNA Regulatory Circuit Controlling Bacterial Denitrification and N2O Emissions
Source: mBio. 2019 Aug 6;10(4):e01165-19. doi: 10.1128/mBio.01165-19 (PMC6686037; doi:10.1128/mBio.01165-19)
Supplement: TABLE S1 [file mBio.01165-19-st001.pdf]

ABL68278	Pden_0161		efflux transporter, RND family, MFP subunit
ABL68748	Pden_0636		Class I peptide chain release factor
ABL68767	Pden_0655		nitrite/sulfite reductase, hemoprotein beta-component, ferrodoxin d
ABL68768	Pden_0656		phosphoadenylylsulfate reductase (thioredoxin)
ABL68769	Pden_0657		conserved hypothetical protein
ABL68770	Pden_0658		Ferredoxin--NADP(+) reductase
ABL68780	Pden_0668		transcriptional regulator, TetR family
ABL68801	Pden_0689		tRNA(Ile)-lysidine synthetase
ABL68903	Pden_0791		HAD-superfamily hydrolase, subfamily IA, variant 3
ABL68993	Pden_0882		6-phosphofructokinase
ABL69027	Pden_0916		transcriptional regulator, TraR/DksA family
ABL69272	Pden_1167		molybdenum ABC transporter, periplasmic molybdate-binding protei
ABL69342	Pden_1237		conserved hypothetical protein
ABL69350	Pden_1245		2-keto-3-deoxy-phosphogluconate aldolase
ABL69484	Pden_1383		hypothetical protein
ABL69786	Pden_1689		globin
ABL69894	Pden_1797		sulfate ABC transporter, ATPase subunit
ABL69895	Pden_1798		sulfate ABC transporter, periplasmic sulfate-binding protein
ABL69896	Pden_1799		sulfate ABC transporter, inner membrane subunit CysT
ABL69897	Pden_1800		sulfate ABC transporter, inner membrane subunit CysW
ABL69924	Pden_1827		heat shock protein Hsp15
ABL69965	Pden_1868		Beta-N-acetylhexosaminidase
ABL69977	Pden_1882		hypothetical protein
ABL69997	Pden_1902		protein of unknown function DUF6, transmembrane
ABL70074	Pden_1982		conserved hypothetical protein
ABL70088	Pden_1996		conserved hypothetical protein n
ABL70227	Pden_2135		conserved hypothetical protein ZK84.1
ABL70287	Pden_2195		Hydroxypyruvate reductase
ABL70415	Pden_2324		FAD-dependent pyridine nucleotide-disulfide oxidoreductase
ABL70561	Pden_2474		ATPase, BadF/BadG/BcrA/BcrD type
ABL70562	Pden_2475	predicted tra	transcriptional regulator, GntR family
ABL70563	Pden_2476		glutamine--fructose-6-phosphate transaminase
ABL70564	Pden_2477		N-acetylglucosamine 6-phosphate deacetylase
ABL70570	Pden_2483	norB	nitric oxide reductase, NorB subunit apoprotein
ABL70571	Pden_2484	norC	nitric oxide reductase, NorC subunit apoprotein
ABL70574	Pden_2487	nirS	dissimilatory nitrite reductase (NO-forming), cytochrome cd1 type ap
ABL70582	Pden_2495		cytochrome d1, heme region
ABL70776	Pden_2689		flavin reductase domain protein, FMN-binding protein
ABL70797	Pden_2713		DNA mismatch repair protein MutL
ABL70825	Pden_2741		hypothetical protein
ABL70932	Pden_2848		Phosphotransferase system, phosphocarrier protein HPr
ABL71485	Pden_3414		conserved hypothetical protein
ABL72124	Pden_4058		protein of unknown function UPF0061
ABL72189	Pden_4125		cation transporter
ABL72283	Pden_4219	nosZ	nitrous oxide reductase apoprotein
ABL72288	Pden_4224		protein of unknown function DUF542, ScdA domain protein

ABL72303	Pden_4239		DNA-O6-methylguanine--protein-cysteine S-methyltransferase
ABL72370	Pden_4306		ABC transporter related protein
ABL72391	Pden_4327		phosphate ABC transporter ATP-binding protein, PhoT family
ABL72433	Pden_4369	predicted tra	transcriptional regulator, LysR family
ABL72439	Pden_4375		ribonucleoside-diphosphate reductase class Ib glutaredoxin subunit
ABL72568	Pden_4504		conserved hypothetical protein
ABL72997	Pden_4937		conserved hypothetical protein
ABL73077	Pden_5017		conserved hypothetical protein

	log2FoldChange		functional catergory
	0.639863	UP	transport
	0.869439	UP	Genetic Information Processing
lomain protein	-1.72719	DOWN	Energy metabolism
	-1.90786	DOWN	Energy metabolism
	-1.45455	DOWN	hypothethical
	-0.8771	DOWN	Energy metabolism
	0.447716	UP	transcriptional regulator
	0.987058	UP	Genetic Information Processing
	0.907832	UP	carbohydrate metabolism
	0.962628	UP	carbohydrate metabolism
	-0.59909	DOWN	transcriptional regulator
n	0.528788	UP	transport
	0.922089	UP	hypothethical
	1.073438	UP	carbohydrate metabolism
	0.613956	UP	hypothethical
	-2.18116	DOWN	Energy metabolism
	-1.66508	DOWN	transport
	-2.05853	DOWN	transport
	-1.64765	DOWN	transport
	-1.63119	DOWN	transport
	1.198005	UP	Genetic Information Processing
	0.688085	UP	carbohydrate metabolism
	1.058049	UP	hypothethical
	0.745342	UP	hypothethical
	1.197134	UP	hypothethical
	0.787668	UP	hypothethical
	0.984826	UP	hypothethical
	0.994124	UP	carbohydrate metabolism
	0.683054	UP	Lipid metabolism
	1.242741	UP	carbohydrate metabolism
	0.808676	UP	transcriptional regulator
	0.794095	UP	carbohydrate metabolism
	0.839942	UP	carbohydrate metabolism
	-1.06656	DOWN	Energy metabolism
	-1.07917	DOWN	Energy metabolism
oprotein	-1.03823	DOWN	Energy metabolism
	-0.80336	DOWN	Energy metabolism
	0.962216	UP	hypothethical
	0.722095	UP	Genetic Information Processing
	-0.75617	DOWN	hypothethical
	0.8047	UP	transport
	0.69627	UP	hypothethical
	0.784683	UP	hypothethical
	0.563168	UP	tranport
	-1.01544	DOWN	Energy metabolism
	-0.94098	DOWN	hypothethical

0.982809	UP	transport
-0.6948	DOWN	transport
-0.41649	DOWN	transport
0.846939	UP	transcriptional regulator
0.57419	UP	hypothethical
0.853746	UP	hypothethical
0.615358	UP	hypothethical
-1.23238	DOWN	hypothethical
